# Personal

**Published:** 1892-06

**Authors:** 


### Personal.

DR. J. B. ANDREWS, Superintendent of the Buffalo State Hospital, was elected president of the Association of Medical Superintendents of American Institutions for the Insane at its recent meeting in Washington.

DR. GILBERT I. CULLEN, editor of the Cincinnati *Medical Journal*, was elected Treasurer of the Ohio State Medical Society, at its recent meeting in Cincinnati.

Dr. M. D. Mann, Professor of Obstetrics and Gynecology in the Buffalo University Medical College, sailed for Europe on the 25th of May, to spend some weeks in Wales and England, expecting to return about the 10th of July. His trip is simply for recreation and rest.

